# Efficacy and safety analysis of immune checkpoint inhibitors combined with chemotherapy in the treatment of advanced triple-negative breast cancer: an umbrella meta-analysis

**DOI:** 10.3389/fonc.2026.1900242

**Published:** 2026-07-21

**Authors:** Wendi Cao, Qingxi Rong, Yingheng Song, Zhijun Ma, Qiuxia Dong

**Affiliations:** 1 Clinical Medicine College, Graduate School of Qinghai University, Xining, Qinghai, China; 2 Department of Medical Oncology, Qinghai Red Cross Hospital, Xining, Qinghai, China; 3 Department of Oncology Surgery, Affiliated Hospital of Qinghai University, Xining, Qinghai, China

**Keywords:** efficacy, immune checkpoint inhibitors, safety, triple-negative breast cancer, umbrella meta-analysis

## Abstract

**Background:**

Triple-negative breast cancer (TNBC) is highly aggressive with poor prognosis and limited treatment options. Immune checkpoint inhibitors (ICIs) combined with chemotherapy have emerged as a promising strategy for advanced TNBC. This umbrella meta-analysis was conducted to comprehensively evaluate the efficacy and safety of this combination regimen.

**Methods:**

We systematically searched PubMed, Web of Science, and Embase for relevant articles up to August 2025. The methodological quality and evidence certainty of the included studies were assessed using AMSTAR−2, GRADE, and a prespecified classification scheme. Publication bias was examined through funnel plots and Egger’s tests, and sensitivity analyses were performed to test result robustness. The appropriate effect model was selected based on heterogeneity.

**Results:**

A total of 11 meta analyses comprising 39,147 patients were included. ICIs combined with chemotherapy significantly improved overall survival (OS, HR = 0.89) and progression-free survival (PFS, HR = 0.81) compared with chemotherapy alone. The objective response rate showed no statistically significant improvement in either programmed cell death ligand-1 (PD-L1)-negative (RR = 1.01) or PD-L1-positive (RR = 1.17) populations. Regarding safety, combination therapy was associated with increased risks of all-grade and grade ≥3 adverse events(AEs), as well as a higher incidence of immune-related adverse events(irAEs). Findings concerning hepatitis require cautious interpretation due to the limited number of studies.

**Conclusions:**

ICIs combined with chemotherapy confers survival benefits in advanced TNBC, though at the cost of increased immune-related toxicity. Given current evidence limitations, future research with more detailed stratification is needed to guide individualized treatment.

**Systematic review registration:**

https://www.crd.york.ac.uk/prospero/, identifier CRD420251168274.

## 1 Introduction

Triple-negative breast cancer (TNBC) accounts for approximately 15%–20% of all breast cancers. Characterized by the absence of estrogen receptor (ER), progesterone receptor (PR), and human epidermal growth factor receptor 2 (HER2) expression, TNBC is regarded as one of the most aggressive subtypes and is associated with the poorest prognosis (1, 2). Globally, the incidence of TNBC has shown a steady increase (3). Compared with other subtypes, TNBC is associated with a lower 5-year survival rate and an elevated propensity for early distant metastasis. This subgroup presents considerable therapeutic challenges and is characterized by a generally poor prognosis (4, 5). Although chemotherapy remains the cornerstone of systemic therapy for TNBC, its efficacy as a single agent is limited in patients with advanced disease (6, 7). Notably, TNBC exhibits a high tumor mutational burden, abundant tumor-infiltrating lymphocytes, and elevated expression of programmed cell death protein-1 (PD-1) and its ligand (PD-L1), together with other immunogenic features that support a theoretical rationale for the use of immune checkpoint inhibitors (ICIs) (8).

ICIs are a class of antitumor therapeutic agents developed via monoclonal antibody technology, with major targets including PD-1/PD-L1 and cytotoxic T-lymphocyte-associated antigen-4 (CTLA-4) (9, 10). PD-1, a key immune inhibitory receptor, is predominantly expressed on the surface of activated T cells (11). Upon binding to PD-L1 on tumor cells or antigen-presenting cells, PD-1 initiates an inhibitory signal cascade, leading to T-cell dysfunction and subsequent tumor immune evasion (12). By specifically blocking the PD-1/PD-L1 axis, ICIs relieve T-cell inhibition, thereby reactivating antitumor immune responses and enhancing the immune system’s recognition and elimination of tumor cells (13, 14). Currently, ICIs constitute one of the cornerstone strategies in tumor immunotherapy (15, 16). Pivotal clinical trials such as IMpassion130 and KEYNOTE−355 have consistently demonstrated that ICI plus chemotherapy significantly prolongs PFS in patients with advanced TNBC (17, 18). In addition, this combination has been shown to increase the ORR to >40%. These findings have positioned this combination as the first-line standard of care for advanced TNBC.

Although ICIs plus chemotherapy provides significant survival benefits in advanced TNBC, the multisystem immune-related adverse events (irAEs) associated with this regimen have become a considerable concern in clinical practice. Previous studies have shown that ICIs may affect multiple organ systems, including the respiratory tract, skin, gastrointestinal tract, and endocrine system (19–21). Among these, although immune-related pneumonitis is relatively uncommon overall, severe cases can progress to respiratory failure. Skin toxicity typically presents as drug eruptions and vitiligo-like changes, while gastrointestinal reactions most frequently present as diarrhea. Among endocrine−related adverse events, thyroid dysfunction is the most frequently reported, affecting approximately 16% of patients for hypothyroidism and 4.9% for hyperthyroidism.

In recent years, several systematic reviews and meta-analyses have examined the efficacy and safety of ICIs plus chemotherapy in TNBC (22–32). However, existing studies have largely concentrated on either a single efficacy endpoint (e.g., PFS or ORR) or specific toxicity events (e.g., irAEs confined to a single organ system). Few have provided a comprehensive assessment that simultaneously addresses survival benefits and multisystem irAEs within a unified evidence framework. This fragmented evidence landscape makes it difficult to form a holistic perspective on the benefit-risk balance of this combination in clinical practice. In view of this, the present study adopts an umbrella meta-analytic approach to systematically synthesize and comprehensively evaluate this topic at a higher evidence level. Efficacy outcomes included overall survival (OS), progression-free survival (PFS), and objective response rate (ORR). The safety assessment covered both any−grade and grade ≥3 adverse events (AEs), with particular emphasis on irAEs, specifically thyroid dysfunction, rash, pneumonitis, and hepatitis.

## 2 Methodology

This study was conducted and reported in strict accordance with the Preferred Reporting Items for Systematic Reviews and Meta-Analyses (PRISMA) 2020 statement (33, 34). The study protocol was prospectively registered with the International Prospective Register of Systematic Reviews (PROSPERO) under registration number CRD420251168274.

### 2.1 Search strategy

We systematically searched PubMed, Web of Science, and Embase for English-language literature on ICIs in TNBC published up to August 2025. These three databases are widely recognized as core sources for clinical medical research evidence. The search strategy combined Medical Subject Headings (MeSH) and free-text keywords, and was structured into eleven components: the first used “Immune Checkpoint Inhibitors” as a MeSH term; the second was “Nivolumab”; the third was “Pembrolizumab”; the fourth was “Camrelizumab”; the fifth was “Atezolizumab”; the sixth was “Avelumab”; the seventh was “Durvalumab”; the eighth was “Ipilimumab”; the ninth used “Triple Negative Breast Neoplasms” as a MeSH term; the tenth was “Systematic review”; and the eleventh was “Meta Analysis”. The first eight components were combined with “OR”, and the ninth to eleventh components were combined with “AND”. The two sets were then merged using “AND” to identify studies relevant to the research topic. The detailed search strategy is provided in **Supplementary Material 1**. For duplicate publications, only the most up-to-date or most complete version was included. In addition, we manually screened the reference lists of relevant articles.

### 2.2 Inclusion and exclusion criteria

The inclusion criteria were systematic reviews and meta-analyses involving adult patients with advanced TNBC treated with ICIs, with no restrictions on gender or ethnicity. However, only studies meeting the following criteria were included:

1. Patients with pathologically confirmed advanced (metastatic or unresectable) TNBC. Studies were excluded if patients had other primary malignancies, or if the study population included non-TNBC patients and their data could not be segregated.
2. Systematic reviews and meta-analyses evaluating the efficacy or safety of ICIs. Basic research, original clinical studies, and systematic reviews without meta-analysis were excluded.
3. The intervention in the experimental group was ICIs combined with standard chemotherapy regimens. Studies employing ICI monotherapy or non-ICI immunotherapies (e.g., chimeric antigen receptor T-cell (CAR-T) therapy and cancer vaccines) were excluded.
4. The control group received either placebo or standard chemotherapy regimens. Any study using ICIs in the control group was excluded.
5. Studies must report at least one pre-specified efficacy outcome (OS, PFS or ORR) or safety outcome (any-grade AEs, grade ≥3 AEs, or specific irAEs). Studies with incomplete data, inability to extract key outcome measures, or duplicate publications were excluded.
6. Studies were published in English.

### 2.3 Screening process

Two investigators independently extracted data using a standardized data extraction form. Any disagreements were first resolved by discussion. If consensus remained elusive, the matter was adjudicated by a third investigator. Extracted content comprised baseline characteristics. These included first author, publication year, number and type of included primary studies, overall sample size, and patient age ranges. Treatment details (regimens for the experimental and control groups) were also recorded. For efficacy, we extracted OS, PFS, and ORR. ORR was further stratified by PD-L1 status based on the combined positive score (CPS), with categories of CPS < 10 and CPS ≥ 10. For safety, we extracted any-grade AEs, grade ≥3 AEs, and specific irAEs.

To systematically assess the potential overlap of primary studies among the included meta-analyses, we constructed a comprehensive citation matrix and calculated the corrected covered area (CCA) according to the methodology proposed by Pieper et al. (35). Based on this framework, the degree of overlap was classified into four categories: slight (0%–5%), moderate (6%–10%), high (11%–15%), and very high (>15%).

### 2.4 Methodological quality assessment

The methodological quality of the included meta-analyses was assessed using the AMSTAR-2 (A MeaSurement Tool to Assess systematic Reviews 2) tool. This instrument consists of 16 items, encompassing 7 critical domains (items 2, 4, 7, 9, 11, 13, 15). Based on the ratings for each item (“yes”, “partial yes”, or “no”), the methodological quality was categorized into four levels: high (no critical domain flaws), moderate (one critical domain flaw), low (more than one critical domain flaw), and critically low (one or more critical flaws) (36).

The GRADE (Grading of Recommendations, Assessment, Development, and Evaluations) approach was used to grade the quality of the evidence body. The assessment covered the domains of risk of bias, imprecision, inconsistency, indirectness, and publication bias. According to the evaluation results, the quality of evidence was classified into four categories: high, moderate, low, and very low (37, 38). In addition, the evidence for each outcome was classified into five categories (Class I, Class II, Class III, Class IV and NS) in accordance with the evidence classification criteria (39, 40).

### 2.5 Publication bias and sensitivity analysis

Publication bias was assessed using a combination of funnel plots and Egger’s linear regression test (41, 42). Initial assessment was performed by visual inspection of funnel plot symmetry, followed by quantitative verification using Egger’s test (significance level α=0.05). A P-value < 0.05 was considered indicative of significant publication bias, while a P-value between 0.05 and 0.10 suggested potential bias due to small-study effects. Additionally, a leave-one-out sensitivity analysis was performed by sequentially excluding individual studies and recalculating the pooled effect size to evaluate the influence of each study on the overall results and to test the robustness of the meta-analysis findings (43).

### 2.6 Data analysis

In the efficacy analysis, OS and PFS were treated as time-to-event outcomes. The summary effect measures for these outcomes were hazard ratios (HRs) with 95% confidence intervals (CIs). ORR being a binary variable, we used odds ratios (ORs) as its summary effect measure. In the safety analysis, the incidence rates of treatment-related AEs and irAEs were likewise binary variables. Depending on the effect measure type, we extracted ORs or relative risks (RRs) from the included studies and performed separate pooled analyses stratified by measure type. More precisely, studies providing ORs contributed to a single pooled OR estimate, whereas those providing RRs contributed to a single pooled RR estimate. For each measure, the summary effect estimate and corresponding 95% CI are presented separately. We did not perform cross-type pooling nor conversion between the two effect measures. Between-study heterogeneity was quantified using both the Cochran Q test and I² statistic (44). Model choice followed a standard rule. When I² ≤ 50% and the Q test yielded P > 0.10, the fixed−effects model (Mantel−Haenszel) was applied (45). Otherwise, the random−effects model (DerSimonian−Laird) was used (46, 47).

All statistical analyses were performed using Stata version 17.0 (Stata Corporation, College Station, TX, USA). Pooled effect estimates and their 95% CIs were presented in forest plots. All statistical tests were two-sided. For heterogeneity testing, a P > 0.10 indicated no significant heterogeneity; for all other tests, a P < 0.05 was considered statistically significant.

## 3 Results

### 3.1 Basic characteristics of the included studies

**Figure 1:**
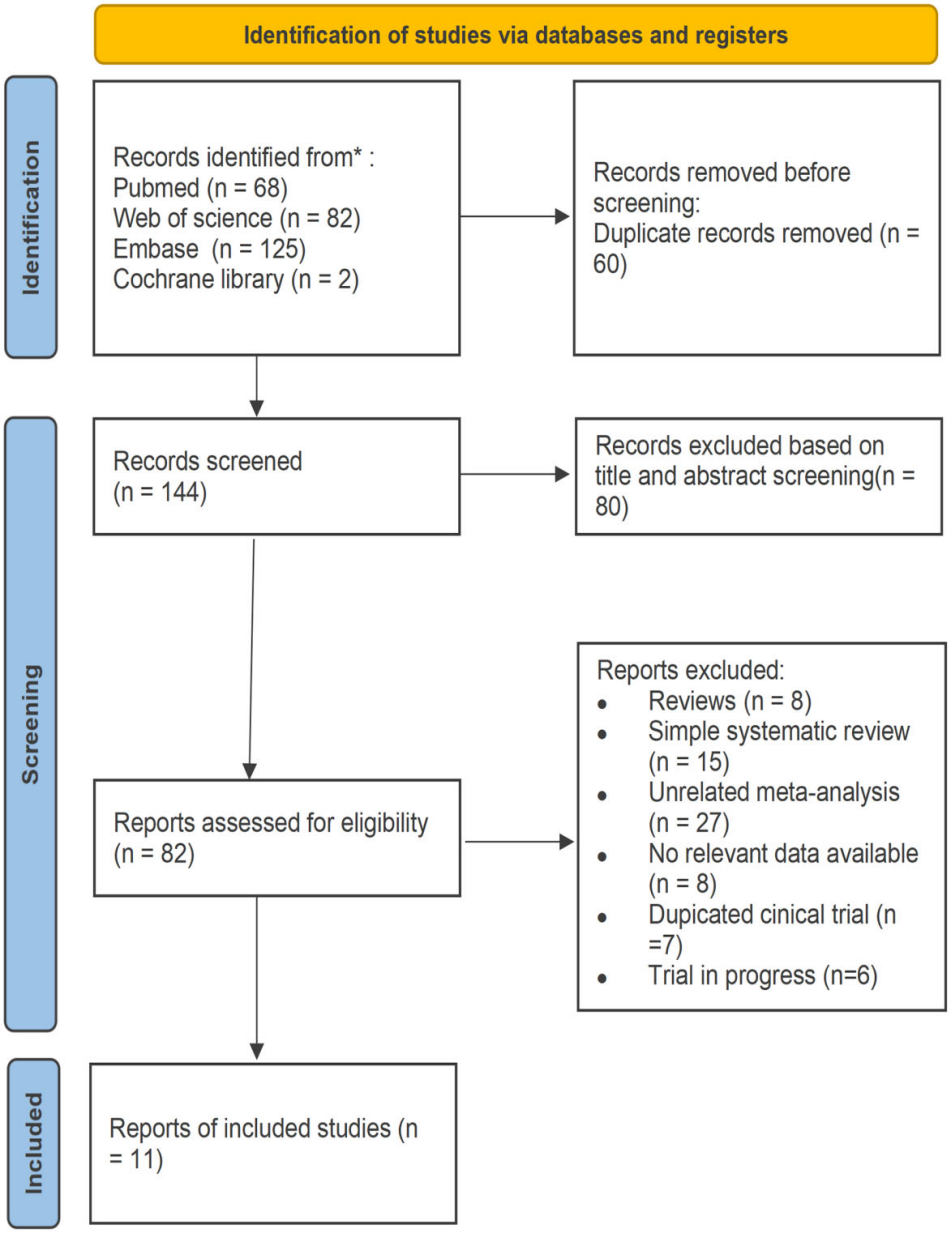
PRISMA flowchart of the literature screening and study selection proces.

A systematic literature search was performed across PubMed (n = 68), Web of Science (n = 82), and Embase (n = 125), which yielded 275 records. Following the removal of 113 duplicate records (**Figure 1**), 82 studies were excluded during title/abstract screening. The remaining 11 meta-analyses were finally included (22–32). The baseline characteristics of the 11 included studies are presented in **Table 1**. Publication years for these studies ranged from 2020 to 2025, with 63.6% (7/11) appearing in 2023 or later. The total pooled sample size across these studies was 39,147 patients. All of the included studies evaluated ICIs combined with chemotherapy in advanced TNBC. The agents examined included pembrolizumab, nivolumab, atezolizumab, durvalumab, and avelumab. When stratified by target, 4 studies focused on PD-L1 inhibitors and 7 focused on PD-1 inhibitors. The frequencies of reported irAEs across studies were as follows: hypothyroidism occurred in 8 studies, hyperthyroidism in 3, rash in 6, pneumonitis in 5, and hepatitis in 2. It is worth noting that some systematic reviews reported more than one irAE type simultaneously. Consequently, we grouped them by irAE type for analysis. The combined study counts across subgroups exceed the total number of included studies.

**Table 1:** Characteristics of the included studies.

First author	Year	Number of studies(Types)	Sample size	Age(Years)	Therapeutic regimen		Efficacy outcome measures		ORR		Safety outcome measures		
					Treatment	Control	OS	PFS	PD-L1-(CPS<10)	PD-L1+(CPS≥10)	All grade	Grade ≥ 3	IrAEs
Bo-Ya Xiao	2020	9(RCT)	1137	50~55	Atezolizumab+CT	CT	HR=0.90(95% CI: 0.81-0.99)	HR=0.85(95% CI: 0.77-0.93)	OR=0.62(95% CI: 0.46-0.85)	OR=0.73(95% CI: 0.56-0.96)	OR=4.57(95% CI: 1.02-5.45)	RR=1.16(95% CI: 0.95-1.42)	Hypothyroidism: OR=7.9(95% CI: 4.0-11.8) Hyperthyroidism: OR=3.2(95% CI: 0.4-6.8) Skin rash: OR=5.0(95% CI: 0.2-10.2)Pneumonitis: OR=2.8(95% CI: 1.4-4.2)
Trujillo Asturias MA	2024	10(RCT)	5945	≥18	Pembrolizumab/ Nivolumab+CT	CT	HR=0.96(95% CI: 0.84–1.09)	HR=0.93(95% CI: 0.64-1.37)	OR=0.81(95% CI: 0.72-0.92)	OR=0.96(95% CI: 0.84-1.09)	OR=1.33(95% CI: 1.03-1.70)	OR=1.09(95% CI: 0.64–1.87)	NR
Khan M	2023	20(RCT)	3962	50~56	Atezolizumab/Durvalumab/Avelumab+CT	CT	HR=0.75(95% CI: 0.61-0.94)	HR=0.88(95% CI: 0.72-1.09)	OR=0.95(95% CI: 0.59-1.52)	OR=1.55(95% CI: 1.25-1.92)	OR=2.89(95% CI: 1.56-5.38)	OR=2.53(95% CI: 1.66-5.53)	Hypothyroidism:OR=2.89(95% CI: 1.56-5.38)
Yonghui Chen	2024	8(RCT)	3338	53~56	Pembrolizumab/ Nivolumab+CT	CT	HR=0.83(95% CI: 0.69-1.00)	HR=0.81(95% CI: 0.75-0.88)	OR=0.71(95% CI: 0.62-0.81)	OR=0.81(95% CI: 0.54-1.23)	OR=2.07(95% CI: 1.38-3.12)	OR=1.09(95% CI: 0.92-1.30)	Hypothyroidism:OR=4.52(95% CI: 2.95-6.94)
Zuxiu Wang	2024	11(RCT)	4314	49~57	Pembrolizumab/ Nivolumab+CT	CT	HR=0.87(95% CI: 0.78-0.96)	HR=0.79(95% CI: 0.70-0.90)	OR=1.07(95% CI: 0.91-1.26)	OR=1.27(95% CI: 1.10-1.46)	RR=1.74(95% CI: 0.97-3.14)	RR=1.36(95% CI: 1.17-1.59)	Pneumonia: RR=3.56(95% CI: 1.78-7.12) Skin rash: RR=1.74(95% CI: 0.97-3.14)
Juan Yang	2024	5(RCT)	3000	53~58.5	Nivolumab/Pembrolizumab/Toripalimab+CT	CT	HR=0.89(95% CI: 0.81-0.97)	HR=0.80(95% CI: 0.73-0.88)	OR=1.35(95% CI: 1.15-1.60)	OR=1.48(95% CI: 1.18-1.86)	RR=1.42(95% CI: 1.15-1.88)	RR=1.09(95% CI: 0.92-1.30)	Hypothyroidism:RR=2.07(95% CI: 1.38-3.12)
Qiao Ji	2021	9(RCT)	4501	47~58	Atezolizumab/Durvalumab+CT	CT	HR=0.86(95% CI: 0.74-0.99)	HR=0.78(95% CI: 0.70-0.86)	OR=1.38(95% CI: 1.01-1.89)	OR=1.92(95% CI: 1.38-2.67)	RR=1.42(95% CI: 1.15-1.83)	RR=1.40(95% CI: 1.13-1.74)	Hypothyroidism:RR=3.82(95% CI: 2.15-6.78) Hyperthyroidism:RR=4.20(95% CI: 2.31-7.65) Pneumonia:RR=4.67(95% CI: 1.32-7.28) Hepatitis:RR=1.66(95% CI: 1.45-4.83) Skin rash:RR=1.17(95% CI: 1.02-1.34)
Ying Wang	2024	7(RCT)	3255	52~59.1	Pembrolizumab/Nivolumab+CT	CT	HR=0.93(95% CI: 0.85-1.01)	HR=0.82(95% CI: 0.74-0.90)	OR=0.61(95% CI: 0.43-0.87)	OR=0.65(95% CI: 0.49-0.86)	OR=1.31(95% CI: 1.09-1.54)	OR=1.31(95% CI: 1.09-1.54)	Hypothyroidism: OR=3.82(95% CI: 2.15-6.78) Hyperthyroidism: OR=4.41(95% CI: 2.01-9.68) Skin rash: OR=2.75(95% CI: 1.62-4.68)
J. Shen	2025	6(RCT)	3105	52~59	Pembrolizumab/Toripalimab+CT	CT	HR=0.87(95% CI: 0.80-0.96)	HR=0.80(95% CI: 0.73-0.87)	OR=1.34(95% CI: 1.15–1.55)	OR=1.47(95% CI: 1.16-1.84)	RR=1.40(95% CI: 1.18-1.66)	RR=1.11(95% CI: 1.04-1.19)	NR
Youran Dai	2024	8(RCT)	4626	48~56	Pembrolizumab/Toripalimab+CT	CT	HR=0.70(95% CI: 0.24-1.80)	HR=0.74(95% CI: 0.47-1.01)	OR=1.25(95% CI: 1.15-1.35)	OR=1.27(95% CI: 1.11-1.46)	OR=1.40(95% CI: 0.95-1.90)	OR=1.20(95% CI: 0.60-2.50)	Hypothyroidism: OR=3.82 (95% CI: 2.01-7.28) Skin rash: OR=2.75(95% CI: 1.52-5.00) Pneumonia: OR=3.60(95% CI: 1.21-10.78)
Sharmni Vishnu	2022	6(RCT)	1964	51~57	Atezolizumab+CT	CT	HR=0.90(95% CI: 0.79-1.01)	HR=0.72(95% CI: 0.59-0.87)	OR=1.70(95% CI: 0.81–3.80)	OR=2.30(95% CI: 1.10–5.20)	RR=1.38(95% CI: 1.13-1.68)	RR=1.03(95% CI: 0.97-1.09)	Hepatitis: RR=1.13(95% CI: 0.94-1.35) Skin rash: RR=1.16(95% CI: 1.02-1.31) Pneumonia: RR=1.36(95% CI: 1.09-1.71) Hypothyroidism: RR=3.77(95% CI: 2.78-5.11)

CT, Chemotherapy; CPS, Combined Positive Score; HR, Hazard Ratio; IrAEs, Immune-Related Adverse Events; NR, Not Reported;OR, Odds Ratio; OS, Overall Survival; ORR, Objective Response Rate; PFS, Progression-Free Survival; RCTs, Randomized Controlled Trials; RR, Relative Risk.

**Figure 2:**
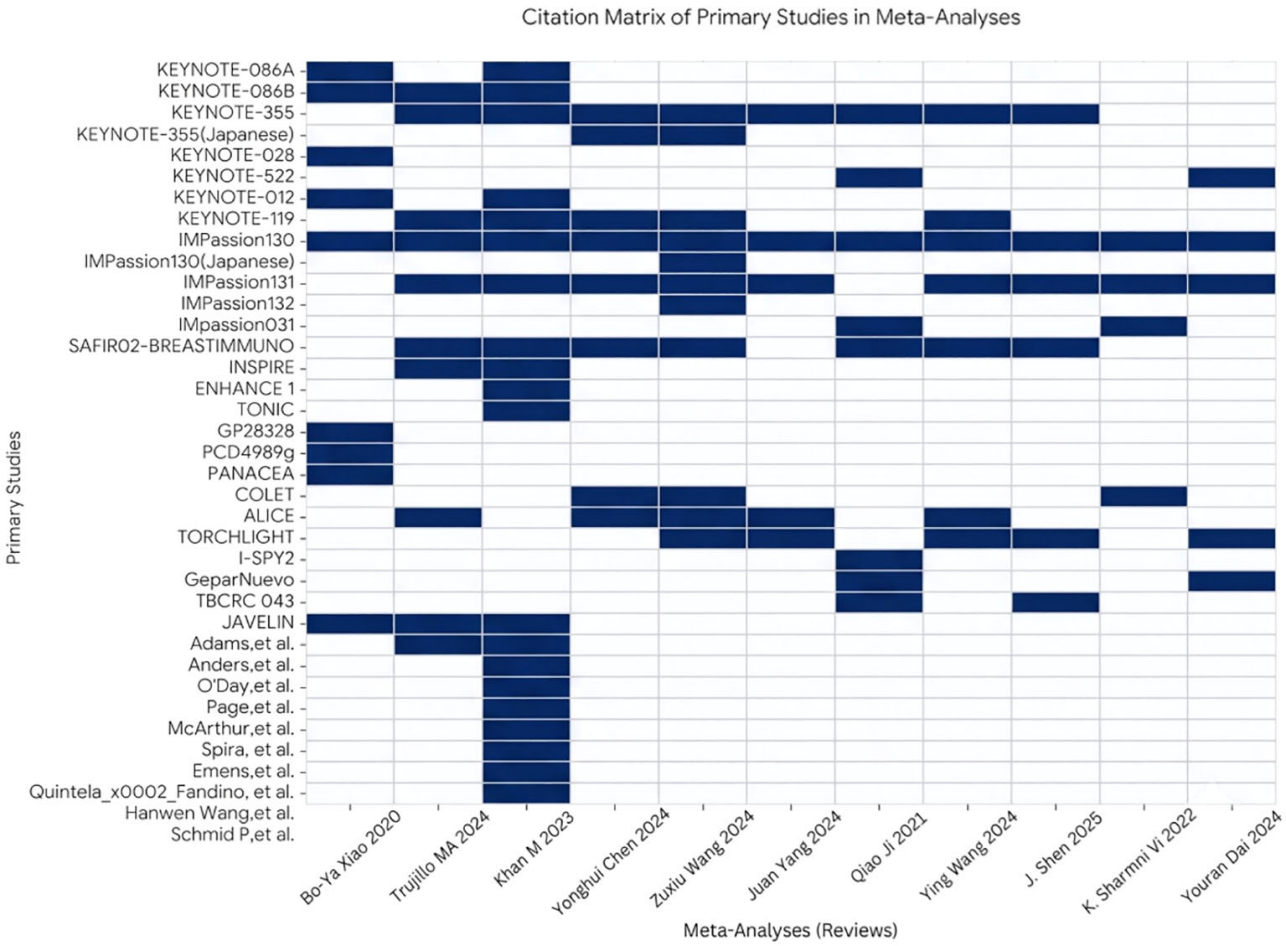
Citation matrix of primary studies included in the meta-analyses.

We constructed a citation matrix for the 11 included meta-analyses to precisely delineate the distribution of primary clinical trials across these studies (**Table 2**). The matrix identified 41 independent primary studies (r = 41), with a cumulative frequency of 99 appearances (N = 99). The CCA was calculated as 14.1% (**Figure 2**). According to the standard thresholds established by Pieper et al., this CCA value indicates a high degree of evidence overlap among the included studies.

**Table 2:** Overlap matrix of primary studies across the included meta-analyses.

Primary study	Bo-Ya Xiao 2020	Trujillo MA 2024	Khan M 2023	Yonghui Chen 2024	Zuxiu Wang 2024	Juan Yang 2024	Qiao Ji 2021	Ying Wang 2024	J. Shen 2025	K. Sharmni Vishnu 2022	Youran Dai 2024
KEYNOTE-086A	1	0	1	0	0	0	0	0	0	0	0
KEYNOTE-086B	1	1	1	0	0	0	0	0	0	0	0
KEYNOTE-355	0	1	1	1	1	1	1	1	1	0	
KEYNOTE-355(Japanese)	0	0	0	1	1	0	0	0	0	0	0
KEYNOTE-028	1	0	0	0	0	0	0	0	0	0	0
KEYNOTE-522	0	0	0	0	0	0	1	0	0	0	1
KEYNOTE-012	1	0	1	0	0	0	0	0	0	0	0
KEYNOTE-119	0	1	1	1	1	0	0	1	0	0	0
IMPassion130	1	1	1	1	1	1	1	1	1	1	1
IMPassion130(Japanese)	0	0	0	0	1	0	0	0	0	0	0
IMPassion131	0	1	1	1	1	1	0	1	1	1	1
IMPassion132	0	0	0	0	1	0	0	0	0	0	0
IMpassion031	0	0	0	0	0	0	1	0	0	1	0
SAFIR02-BREASTIMMUNO	0	1	1	1	1	0	1	1	1	0	0
INSPIRE	0	1	1	0	0	0	0	0	0	0	0
ENHANCE 1	0	0	1	0	0	0	0	0	0	0	0
TONIC	0	0	1	0	0	0	0	0	0	0	0
GP28328	1	0	0	0	0	0	0	0	0	0	0
PCD4989g	1	0	0	0	0	0	0	0	0	0	0
PANACEA	1	0	0	0	0	0	0	0	0	0	0
COLET	0	0	0	1	1	0	0	0	0	1	0
ALICE	0	1	0	1	1	1	0	1	0	0	0
TORCHLIGHT	0	0	0	0	1	1	0	1	1	0	1
I-SPY2	0	0	0	0	0	0	1	0	0	0	0
GeparNuevo	0	0	0	0	0	0	1	0	0	0	1
TBCRC 043	0	0	0	0	0	0	1	0	1	0	0
JAVELIN	1	1	1	0	0	0	0	0	0	0	0
Adams,et al.	0	1	1	0	0	0	0	0	0	0	0
Anders,et al.	0	0	1	0	0	0	0	0	0	0	0
O'Day,et al.	0	0	1	0	0	0	0	0	0	0	0
Page,et al.	0	0	1	0	0	0	0	0	0	0	0
McArthur,et al.	0	0	1	0	0	0	0	0	0	0	0
Spira, et al.	0	0	1	0	0	0	0	0	0	0	0
Emens,et al.	0	0	1	0	0	0	0	0	0	0	0
QuintelaFandino, et al.	0	0	1	0	0	0	0	0	0	0	0
Hanwen Wang,et al.	0	0	0	0	0	0	0	0	0	1	0
Loibl S,et al.	0	0	0	0	0	0	0	0	0	0	1
Gianni L,et al.	0	0	0	0	0	0	0	0	0	0	1
Cortes J,et al.	0	0	0	0	0	0	0	0	0	0	1
Tolaney SM,et al.	0	0	0	0	0	0	1	0	0	0	0
Schmid P,et al.	0	0	0	0	0	0	0	0	0	1	0

Overlap matrix of primary studies across the included meta-analyses. In the matrix, '1' indicates that the primary study is cited in the corresponding meta-analysis, and '0' indicates that it is not cited.

### 3.2 Methodological and evidence quality assessment

The methodological quality of the 11 included meta-analyses was assessed using the AMSTAR-2 tool (**Supplementary Material 2**). The results showed that 5 were of moderate quality (Khan 2023, Zuxiu Wang 2024, Juan Yang 2024, Qiao Ji 2021, Youran Dai 2024), 4 were of low quality (Bo-Ya Xiao 2020, Ying Wang 2024, J. Shen 2025, Sharmni Vishnu 2022), and 2 were of critically low quality (Trujillo Asturias MA 2024, Yonghui Chen 2024). The two critically low quality reviews each had at least one critical flaw, such as failure to preregister the protocol or report protocol deviations, inadequate assessment of the risk of bias in primary studies, insufficient consideration of bias when interpreting results, or lack of reasonable explanation for inter-study heterogeneity.

Evidence quality was assessed using the GRADE system (**Supplementary Material 3**). Among the 11 studies, 3 were rated as high quality (27.3%; Juan Yang 2024, J. Shen 2025, Youran Dai 2024), 5 as moderate quality (45.5%), 1 as low quality (9.1%; Trujillo Asturias MA 2024), and 2 as very low quality (18.2%; Bo−Ya Xiao 2020, Yonghui Chen 2024). According to the evidence classification criteria, the total number of participants across all comparisons did not exceed 20,000. Therefore, all results were classified as Class IV evidence.

Overall, the quality of evidence from the meta-analyses included in this study was not high. This phenomenon may be related to heterogeneity across studies and potential publication bias. Therefore, the strength of this conclusion is limited. Caution should be exercised when interpreting these findings in clinical decision-making, considering the uncertainty associated with low-quality evidence.

### 3.3 Risk of bias and sensitivity analysis

Publication bias was evaluated using both qualitative assessment of funnel plots (**Supplementary Material 4**) and quantitative analysis via Egger’s regression test (**Supplementary Material 5**). For efficacy outcomes (OS, PFS, and the PD−L1−negative and −positive subgroups), the funnel plots were largely symmetrical, and all Egger’s tests were not statistically significant (P > 0.05). For safety outcomes, the funnel plots for any−grade AEs, grade ≥3 AEs, hypothyroidism, and rash showed varying degrees of asymmetry. Egger’s tests correspondingly suggested potential publication bias. For hyperthyroidism (n = 3), pneumonitis (n = 5), and hepatitis (n = 2), the limited number of included studies precluded meaningful assessment of funnel plot symmetry. Notably, Egger’s test could not be performed for hepatitis.

Leave−one−out sensitivity analysis was performed to assess the robustness of the pooled results (**Supplementary Material 6**). Sequentially excluding each study did not change the direction of the pooled estimates for OS, PFS, or the PD−L1−negative and −positive subgroups. Similarly, no directional reversal occurred in the pooled estimates for any−grade AEs, grade ≥3 AEs, hypothyroidism, and rash. In contrast, the pooled estimates for pneumonitis and hyperthyroidism had relatively wide confidence intervals, indicating less robust stability. Given that only two studies were included for hepatitis, findings for this outcome are highly dependent on individual studies and should be interpreted with caution.

### 3.4 Impact of ICIs plus chemotherapy on OS and PFS in advanced TNBC and subgroup analyses

**Figure 3:**
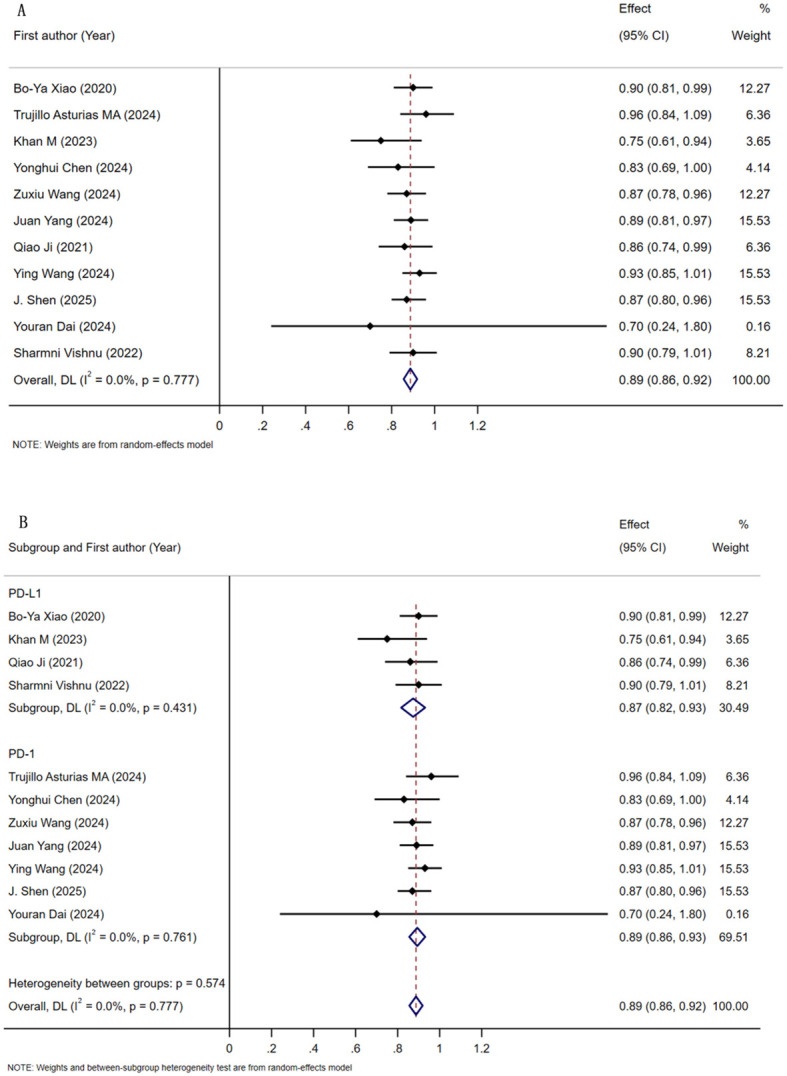
Forest plots of OS in patients with advanced TNBC treated with ICIs plus chemotherapy. **(A)** Overall population; **(B)** Subgroup analyses stratified by ICI type (PD-1 vs. PD-L1 inhibitors).

Forest plot analysis demonstrated that ICIs plus chemotherapy significantly improved OS compared with chemotherapy alone in patients with advanced TNBC (HR = 0.89, 95% CI: 0.86–0.92). This corresponds to an 11% reduction in the risk of death (high quality; Class IV), and no heterogeneity was detected across studies (I² = 0.0%, **Figure 3A**). Subgroup analysis (**Figure 3B**) showed that the pooled HR was 0.87 (95% CI: 0.82–0.93) for the PD−L1 subgroup (4 studies) and 0.89 (95% CI: 0.86–0.93) for the PD−1 subgroup (7 studies). The between−subgroup difference was not statistically significant (P = 0.574). Although two low−weight studies had individual effect estimates whose CIs crossed 1.0, their inclusion did not materially alter the overall pooled estimate.

**Figure 4:**
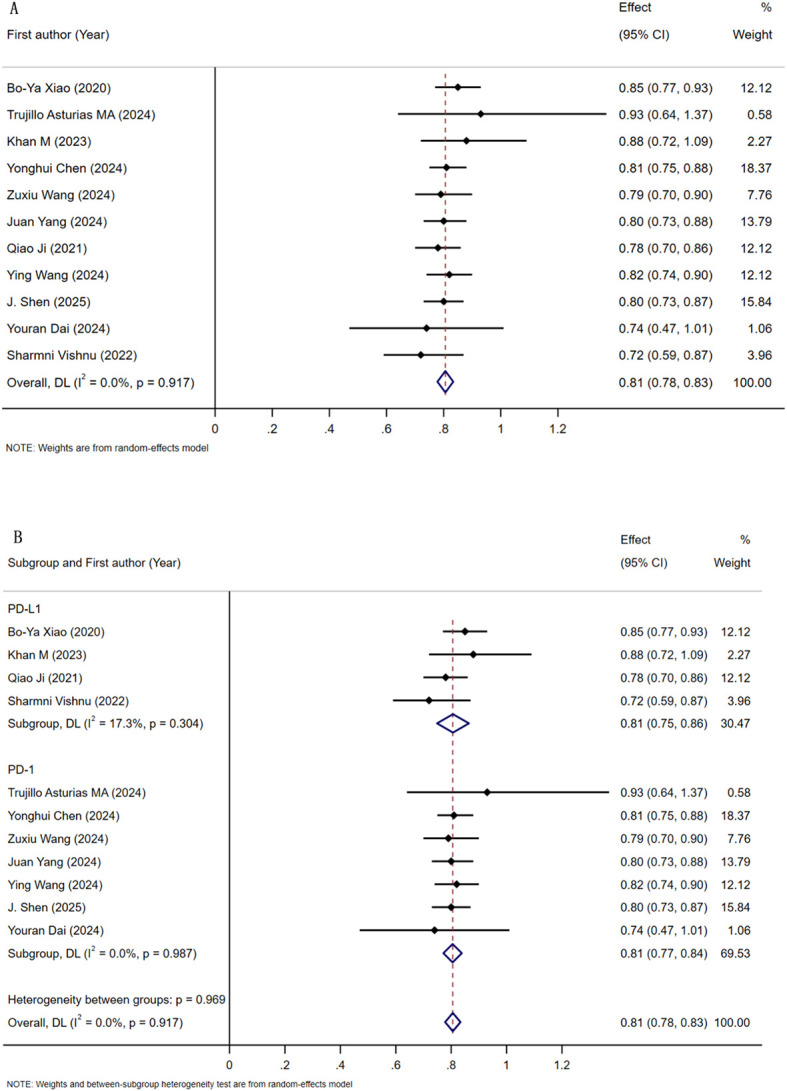
Forest plots of PFS in patients with advanced TNBC treated with ICIs plus chemotherapy. **(A)** Overall population; **(B)** Subgroup analyses stratified by ICI type (PD-1 vs. PD-L1 inhibitors).

For PFS, the pooled HR was 0.81 (95% CI: 0.78–0.83, **Figure 4A**), which corresponds to a 19% risk reduction for progression or death (high quality; Class IV). In subgroup analysis (**Figure 4B**), the pooled HR for the PD−L1 subgroup was 0.81 (95% CI: 0.75–0.86), and heterogeneity was mild (I² = 17.3%, P = 0.304). For the PD−1 subgroup, the pooled HR was 0.81 (95% CI: 0.77–0.84), and no heterogeneity was observed (I² = 0.0%, P = 0.987). The between−subgroup difference remained statistically non−significant (P = 0.969). One study (weight 0.58%) had a CI that crossed 1.0, but this did not affect the overall estimate.

In summary, ICIs plus chemotherapy conferred statistically significant benefits in terms of both OS and PFS for patients with advanced TNBC, and no significant between−subgroup differences were observed between the PD−L1 and PD−1 subgroups.

### 3.5 Impact of ICIs plus chemotherapy on ORR in advanced TNBC and subgroup analyses

**Figure 5:**
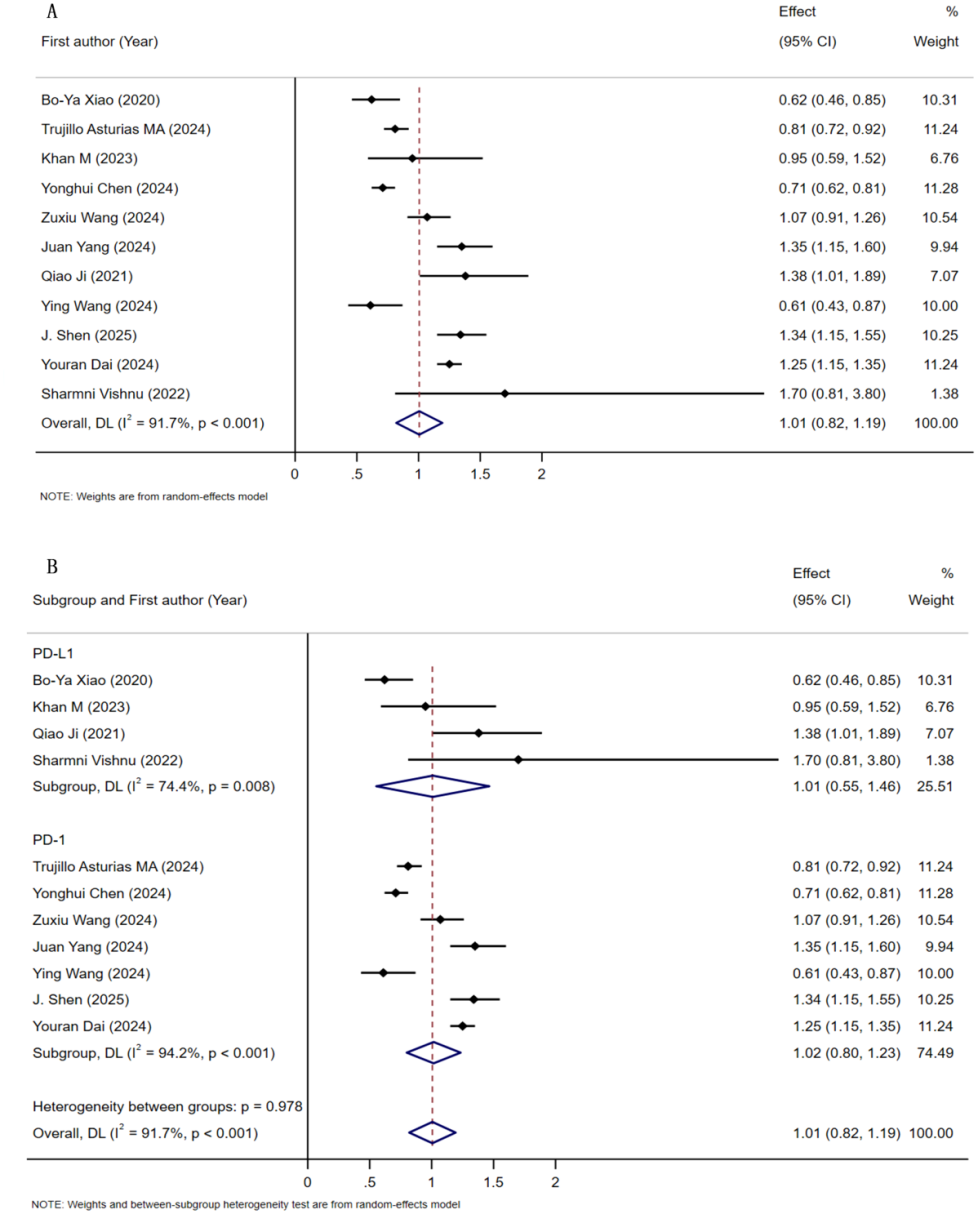
Forest plots of ORR in the PD-L1−negative (CPS < 10) population treated with ICIs plus chemotherapy. **(A)** Overall population; **(B)** Subgroup analyses stratified by ICI type (PD-1 vs. PD-L1 inhibitors).

In the PD−L1−negative (CPS < 10) population, the pooled OR was 1.01 (95% CI: 0.82–1.19), which was not statistically significant (moderate quality; Class IV), and heterogeneity was high across studies (I² = 91.7%, **Figure 5A**). Subgroup analysis (**Figure 5B**) showed pooled ORs of 1.01 (95% CI: 0.55–1.46) for the PD−L1 inhibitor subgroup and 1.02 (95% CI: 0.80–1.23) for the PD−1 inhibitor subgroup. The between−subgroup difference was not statistically significant (P = 0.385).

**Figure 6:**
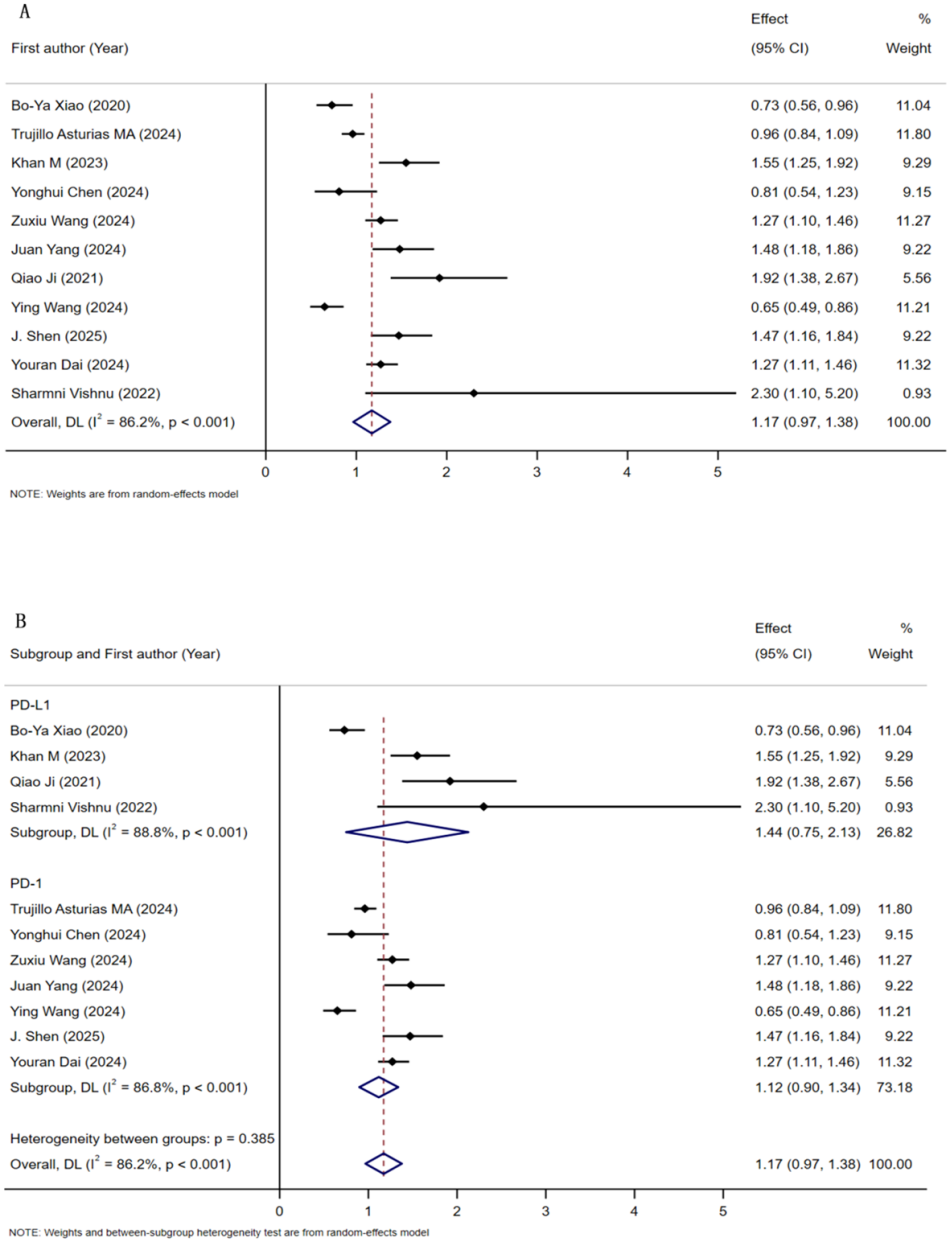
Forest plots of ORR in the PD-L1−positive (CPS ≥ 10) population treated with ICIs plus chemotherapy. **(A)** Overall population; **(B)** Subgroup analyses stratified by ICI type (PD-1 vs. PD-L1 inhibitors).

In the PD−L1−positive (CPS ≥ 10) population (**Figure 6A**), the pooled OR was 1.17 (95% CI: 0.97–1.38), which was not statistically significant (moderate quality; Class IV). Subgroup analysis (**Figure 6B**) revealed pooled ORs of 1.44 (95% CI: 0.75–2.13) for the PD−L1 inhibitor subgroup and 1.12 (95% CI: 0.90–1.34) for the PD−1 inhibitor subgroup; both subgroups exhibited high within−group heterogeneity. The substantial between−study heterogeneity observed across subgroups may be attributable to differences among the original meta−analyses in terms of ICIs type, treatment line, chemotherapy backbone, and PD−L1 assay methods.

In summary, ICIs plus chemotherapy did not significantly improve ORR in patients with advanced TNBC, regardless of PD−L1 expression level.

### 3.6 Impact of ICIs plus chemotherapy on treatment-related AEs in advanced TNBC and subgroup analyses

**Figure 7:**
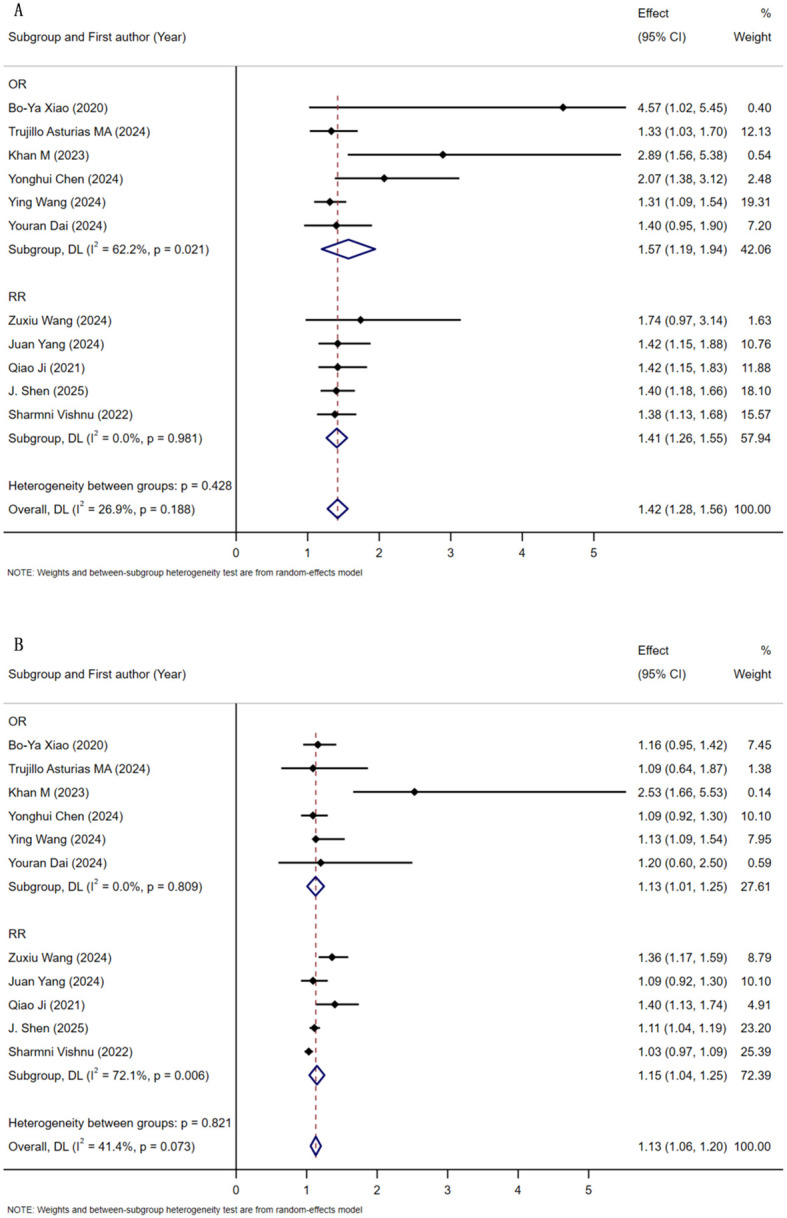
Forest plots of treatment-related AEs. **(A)** All-grade AEs; **(B)** Grade ≥ 3 AEs.

Compared with chemotherapy alone, the pooled effect estimate for any−grade AEs was 1.42 (95% CI: 1.28–1.56), which was statistically significant (moderate quality; Class IV), and heterogeneity was low across studies (I² = 26.9%, **Figure 7A**). Specifically, the pooled RR in the OR subgroup was 1.57 (95% CI: 1.19–1.94), and within−group heterogeneity was moderate (I² = 62.2%, P = 0.021). For the RR subgroup, the pooled RR was 1.41 (95% CI: 1.26–1.55), and no within−group heterogeneity was observed (I² = 0.0%, P = 0.981).

For grade ≥3 AEs, the pooled effect estimate was 1.13 (95% CI: 1.06–1.20), which was statistically significant (moderate quality; Class IV, **Figure 7B**). In the OR subgroup, the pooled RR was 1.13 (95% CI: 1.01–1.25), and no within−group heterogeneity was detected (I² = 0.0%, P = 0.809). In the RR subgroup, the pooled RR was 1.15 (95% CI: 1.04–1.25), and within−group heterogeneity was substantial (I² = 72.1%, P = 0.006). The between−subgroup difference was not statistically significant (P = 0.821).

In summary, ICIs plus chemotherapy was associated with an increased risk of both any−grade and grade ≥3 treatment−related AEs in patients with advanced TNBC.

### 3.7 Impact of ICIs plus chemotherapy on irAEs in advanced TNBC and subgroup analyses

**Figure 8:**
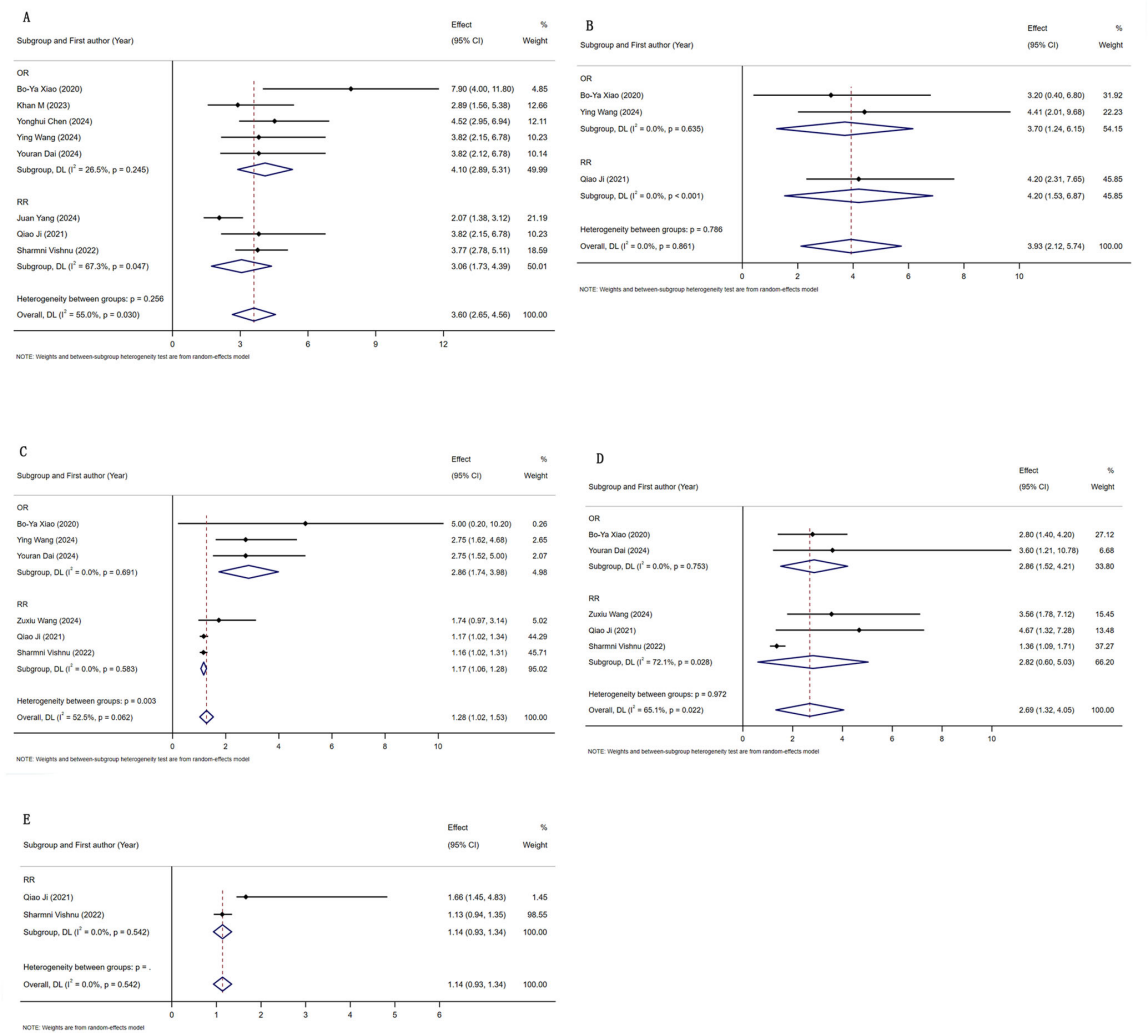
Forest plots of irAEs. **(A)** Hypothyroidism; **(B)** Hyperthyroidism; **(C)** Rash; **(D)** Pneumonitis; **(E)** Hepatitis.

Forest plot analysis demonstrated that ICIs plus chemotherapy was associated with an increased risk of hypothyroidism (**Figure 8A**), hyperthyroidism (**Figure 8B**), rash (**Figure 8C**), and pneumonitis (**Figure 8D**). The pooled estimates were 3.60 (95% CI: 2.65–4.56), 3.93 (95% CI: 2.12–5.74), 1.28 (95% CI: 1.02–1.53), and 2.69 (95% CI: 1.32–4.05), and all were statistically significant (moderate quality; Class IV). Heterogeneity across studies varied by outcome: it was moderate for hypothyroidism (I² = 55.0%, P = 0.030), absent for hyperthyroidism (I² = 0.0%, P = 0.861), moderate for rash (I² = 52.5%, P = 0.062), and substantial for pneumonitis (I² = 65.1%, P = 0.022). Notably, only two studies were available for hepatitis (RR = 1.14, 95% CI: 0.93–1.34, **Figure 8E**), and the difference was not statistically significant (moderate quality; Class IV). Based on the currently limited data, a definitive association between ICIs plus chemotherapy and hepatitis cannot be established.

Stratified subgroup analysis by effect measure type showed the following patterns. For hypothyroidism, the pooled estimate was 4.10 (95% CI: 2.89–5.31) in the OR subgroup and 3.06 (95% CI: 1.73–4.39) in the RR subgroup. For hyperthyroidism, the corresponding values were 3.70 (95% CI: 1.24–6.15) and 4.20 (95% CI: 1.53–6.87). For pneumonitis, the estimates were 2.86 (95% CI: 1.52–4.21) and 2.82 (95% CI: 0.60–5.03). For rash, the estimates were 2.86 (95% CI: 1.74–3.98) and 1.17 (95% CI: 1.06–1.28). The between−subgroup difference was statistically significant (P = 0.003). This suggests that the choice of effect measure may be a source of heterogeneity for rash.

## 4 Discussion

This study used an umbrella meta−analysis approach to aggregate the existing evidence and appraise the certainty of this evidence regarding the efficacy and safety of ICIs combined with chemotherapy in advanced TNBC. A total of 11 meta−analyses were included, covering 99 primary studies and 39,147 patients, which represents a large−scale evidence synthesis in this field. In contrast to traditional meta−analyses that examine only a single outcome, the strengths of this study are threefold: First, it re−synthesizes and cross−compares evidence at the meta−analysis level. This quantifies benefit magnitude and evidence reliability across different therapeutic targets, clinical subgroups, and outcome measures within a unified analytical framework. Second, it integrates the AMSTAR−2 tool, the GRADE system, and standardized evidence grading to enable concurrent interpretation of pooled estimates, methodological quality, and evidence certainty. Third, through a prospective design, comprehensive searches of multiple databases, and independent screening and data extraction by two reviewers, it minimizes the risk of selection bias affecting the conclusions.

Chemotherapy remains a cornerstone of treatment for advanced TNBC, though its long−term survival benefit as a single agent is modest. ICIs combined with chemotherapy has become a key strategy to improve outcomes in advanced TNBC. Our results showed that ICIs combined with chemotherapy improved both OS and PFS in patients with advanced TNBC compared with chemotherapy alone. These findings are consistent with those of previous meta−analyses (22, 25–30). Subgroup analysis suggested that both PD−1 and PD−L1 inhibitors combined with chemotherapy provided consistent survival benefits. However, the pooled OS estimate for PD−1 inhibitors showed lower between−study heterogeneity. This efficacy difference may stem from their distinct mechanisms of action (48): PD−1 inhibitors block both the PD−1/PD−L1 and PD−1/PD−L2 pathways, whereas PD−L1 inhibitors act only on the PD−1/PD−L1 axis (49, 50). This broader pathway blockade may modestly influence both antitumor immunity and immune homeostasis. Additionally, different chemotherapies differ in their ability to reshape the tumor immune microenvironment, so regimen choice could confound the observed efficacy of combination therapy. Relative to standard paclitaxel, nab−paclitaxel may more robustly promote the accumulation of stem−like CD8^+^ T cells and follicular helper T cells, enhancing antitumor immunity and boosting the synergy of the combination (48, 51).

For ORR, our pooled results showed no significant advantage for ICIs plus chemotherapy over chemotherapy alone, irrespective of PD−L1 status (CPS < 10 vs. ≥ 10). Immunologically, PD−L1−high tumors (CPS ≥ 10) typically exhibit active intratumoral T−cell infiltration (52, 53). Theoretically, chemotherapy−induced immunogenic cell death (ICD) added to ICIs could produce potent synergistic antitumor effects (54, 55). However, our ORR findings did not bear out this theoretical benefit in practice. PD−L1−low tumors tend to be immunologically “cold,” with defective antigen presentation and sparse intratumoral immune cells (56). Combination therapy may not completely overcome the immune−suppressed state (57); this explains why ORR remained unchanged in these patients.

Overactivation of the immune system by ICIs drives irAEs (58). Our findings show that ICIs plus chemotherapy raises the risk of both any−grade and grade ≥3 AEs versus chemotherapy alone, with thyroid dysfunction, rash, and pneumonitis as the most frequent irAEs (59, 60). This pattern matches the observations of Yang HR and colleagues (61). Clinically, clinicians should monitor thyroid function, skin changes, and respiratory symptoms throughout treatment for early recognition and timely intervention. Mechanistically, thyroid dysfunction arises from cross−reactive T−cell attacks on self−antigens like thyroid peroxidase and thyroglobulin (62, 63). Skin toxicity results from cytotoxic T−cell damage to epidermal keratinocyte self−antigens (60). Pneumonitis, by contrast, involves T−cell cross−recognition of shared epitopes on tumor cells and alveolar epithelial cells, triggering localized immune inflammation (21). Notably, only two meta−analyses contributed to our hepatitis analysis; thus, no firm link between ICIs plus chemotherapy and hepatitis can be inferred. Larger and better−designed studies are needed to confirm these findings.

Several limitations should be acknowledged. First, there is considerable overlap of primary studies among the included meta-analyses. This is largely attributable to the fact that all contemporary meta-analyses in this field have consistently focused on the same pivotal phase III trials (e.g., IMpassion130, KEYNOTE-355, and IMpassion131) as their core benchmarks, reflecting the high concentration of evidence in advanced TNBC research. Such non-independent data structure may potentially inflate the pooled effect estimates and increase the risk of false-positive findings, thereby compromising the reliability of subgroup analyses. Second, aggregated data prevented detailed stratification by ICIs type, treatment line, chemotherapy backbone, or PD−L1 assay. These variables are likely sources of the heterogeneity observed. Third, the included meta−analyses were often methodologically weak. Umbrella reviews are only as strong as their component studies; poor−quality inputs can bias the estimates and lower our confidence in the results. Fourth, several safety outcomes showed possible publication bias, and a few could not be tested quantitatively owing to sparse data. Thus, the safety estimates may not reflect true risks, so toxicity data need careful reading.

## 5 Conclusion

In summary, ICIs combined with chemotherapy improved OS and PFS in patients with advanced TNBC. This benefit was consistent across different PD−L1 expression levels and immunotherapy target subgroups. However, the combination regimen also increased the risk of overall AEs and specific irAEs. Therefore, the benefit−risk balance should be weighed in clinical practice, and safety monitoring should be reinforced. Owing to limitations including the methodological quality of the primary studies, publication bias, between−study heterogeneity, and study overlap, the conclusions of this study should be interpreted with caution. Future research should include more well−designed randomized controlled trials with standardized baseline characteristics, as well as high−quality meta−analyses that incorporate detailed information on chemotherapy regimens and biomarker assessments. Such efforts will help to better define the patient population that may benefit from combination therapy and to optimize safety management strategies.

## Data Availability

The original contributions presented in the study are included in the article/**Supplementary Material**. Further inquiries can be directed to the corresponding author.
