# Supplementary material for: Efficacy and safety analysis of immune checkpoint inhibitors combined with chemotherapy in the treatment of advanced triple-negative breast cancer: an umbrella meta-analysis
Source: Front Oncol. 2026 Jul 21;16:1900242. doi: 10.3389/fonc.2026.1900242 (PMC13433306; doi:10.3389/fonc.2026.1900242)
Supplement: Supplementary file 1 [file Table1.docx]

**Supplementary Materials**

**Supplementary Material 1**

**Search terms and themes used in the search strategy**

| **Immune**  **Checkpoint Inhibitors** | - Checkpoint Inhibitors, Immune - Immune Checkpoint Blockers - Checkpoint Blockers, Immune - Immune Checkpoint Inhibitor - Checkpoint Inhibitor, Immune - CTLA-4 Inhibitors - CTLA 4 Inhibitors - Cytotoxic T-Lymphocyte-Associated Protein 4 Inhibitors - Cytotoxic T Lymphocyte Associated Protein 4 Inhibitors - Cytotoxic T-Lymphocyte-Associated Protein 4 Inhibitor - Cytotoxic T Lymphocyte Associated Protein 4 Inhibitor - CTLA-4 Inhibitor - CTLA 4 Inhibitor - PD-1 Inhibitors - PD 1 Inhibitors - Programmed Cell Death Protein 1 Inhibitor - Programmed Cell Death Protein 1 Inhibitors - PD-1 Inhibitor - Inhibitor, PD-1 - PD 1 Inhibitor - Immune Checkpoint Blockade - Checkpoint Blockade, Immune - Immune Checkpoint Inhibition - Checkpoint Inhibition, Immune - PD-L1 Inhibitors - PD L1 Inhibitors - Programmed Death-Ligand 1 Inhibitors - Programmed Death Ligand 1 Inhibitors - PD-L1 Inhibitor - PD L1 Inhibitor - PD-1-PD-L1 Blockade - Blockade, PD-1-PD-L1 - PD 1 PD L1 Blockade |
| --- | --- |
| **Nivolumab** | - MDX-1106 - MDX1106 - MDX 1106 - Opdivo - BMS-936558 - BMS936558 - BMS 936558 - ONO-4538 - ONO4538 - ONO 4538 |
| **Pembrolizumab** | - MK-3475 - Keytruda - lambrolizumab - SCH-900475 |
| **Camrelizumab** | - SHR-1210 - SHR 1210 - carrelizumab |
| **Atezolizumab** | - immunoglobulin G1, anti-(human CD antigen CD274) (human monoclonal MDPL3280a heavy chain), disulfide with human monoclonal MDPL3280a kappa-chain, dimer - anti-PDL1 - MPDL3280A - MPDL-3280A - Tecentriq - RG7446 - RG-7446 |
| **Avelumab** | - MSB0010718C - MSB-0010718C - bavencio - MSB-0010682 - MSB0010682 |
| **Durvalumab** | - MEDI4736 - MEDI-4736 - Imfinzi |
| **Ipilimumab** | - Anti-CTLA-4 MAb Ipilimumab - Anti CTLA 4 MAb Ipilimumab - Ipilimumab, Anti-CTLA-4 MAb - MDX 010 - MDX-010 - MDX010 - MDX-CTLA-4 - MDX CTLA 4 - Yervoy |
| **Triple Negative**  **Breast Neoplasms** | - ER-Negative PR-Negative HER2-Negative Breast Cancer - ER Negative PR Negative HER2 Negative Breast Cancer - ER-Negative PR-Negative HER2-Negative Breast Neoplasms - ER Negative PR Negative HER2 Negative Breast Neoplasms - Triple Negative Breast Cancer - Triple-Negative Breast Cancer - Breast Cancers, Triple-Negative - Breast Cancer, Triple-Negative - Triple-Negative Breast Cancers - Triple-Negative Breast Neoplasm - Breast Neoplasms, Triple-Negative - Breast Neoplasm, Triple-Negative - Triple Negative Breast Neoplasm - Triple-Negative Breast Neoplasms |
| **Systematic review** |  |
| **Meta-analysis** |  |

**Supplementary Material 1**

**Search strings for each database**

| **Database** | **Search string** |
| --- | --- |
| **PubMed** | (((((((((((((((((((((((((((Immune Checkpoint Inhibitors[MeSH Terms]) OR (Checkpoint Inhibitor*, Immune[Title/Abstract])) OR (Immune Checkpoint Blocker*[Title/Abstract])) OR (Checkpoint Blocker*, Immune[Title/Abstract])) OR (Immune Checkpoint Inhibitor*[Title/Abstract])) OR (CTLA*4 Inhibitor*[Title/Abstract])) OR (Cytotoxic T*Lymphocyte*Associated Protein 4 Inhibitor*[Title/Abstract])) OR (PD*1 Inhibitor*[Title/Abstract])) OR (Programmed Cell Death Protein 1 Inhibitor*[Title/Abstract])) OR (Inhibitor, PD-1[Title/Abstract])) OR (PD 1 Inhibitor[Title/Abstract])) OR (Immune Checkpoint Blockade[Title/Abstract])) OR (Checkpoint Blockade, Immune[Title/Abstract])) OR (Immune Checkpoint Inhibition[Title/Abstract])) OR (Checkpoint Inhibition, Immune[Title/Abstract])) OR (PD*L1 Inhibitor*[Title/Abstract])) OR (Programmed Death*Ligand 1 Inhibitor*[Title/Abstract])) OR (PD*1*PD*L1 Blockade[Title/Abstract])) OR (Blockade, PD-1-PD-L1[Title/Abstract])) OR (((((Nivolumab[MeSH Terms]) OR (MDX 1106[Title/Abstract])) OR (Opdivo[Title/Abstract])) OR (BMS 936558[Title/Abstract])) OR (ONO 4538[Title/Abstract]))) OR ((((((Pembrolizumab[MeSH Terms]) OR (MK-3475[Title/Abstract])) OR (Keytruda[Title/Abstract])) OR (lambrolizumab[Title/Abstract])) OR (SCH-900475[Title/Abstract])))) OR (((Camrelizumab[MeSH Terms]) OR (SHR 1210[Title/Abstract])) OR (carrelizumab[Title/Abstract]))) OR (((((Atezolizumab[MeSH Terms]) OR (anti-PDL1[Title/Abstract])) OR (MPDL*3280A[Title/Abstract])) OR (Tecentriq[Title/Abstract])) OR (R-G7446[Title/Abstract]))) OR ((((Avelumab[MeSH Terms]) OR (MSB 0010718C[Title/Abstract])) OR (bavencio[Title/Abstract])) OR (MSB 0010682[Title/Abstract]))) OR (((Durvalumab[MeSH Terms]) OR (MEDI*4736[Title/Abstract])) OR (Imfinzi[Title/Abstract]))) OR ((((((((((Ipilimumab[MeSH Terms]) OR (Anti-CTLA-4 MAb Ipilimumab[Title/Abstract])) OR (Anti CTLA 4 MAb Ipilimumab[Title/Abstract])) OR (Ipilimumab, Anti-CTLA-4 MAb[Title/Abstract])) OR (MDX 010[Title/Abstract])) OR (MDX-010[Title/Abstract])) OR (MDX010[Title/Abstract])) OR (MDX-CTLA-4[Title/Abstract])) OR (MDX CTLA 4[Title/Abstract])) OR (Yervoy[Title/Abstract]))) AND ((((((((Triple Negative Breast Neoplasms[MeSH Terms]) OR (ER Negative PR-Negative HER2*Negative Breast Cancer*[Title/Abstract])) OR (ER Negative PR*Negative HER2*Negative Breast Neoplasm*[Title/Abstract])) OR (Triple*Negative Breast Cancer[Title/Abstract])) OR (Breast Cancer*, Triple-Negative[Title/Abstract])) OR (Triple-Negative Breast Cancer*[Title/Abstract])) OR (Triple*Negative Breast Neoplasm*[Title/Abstract])) OR (Breast Neoplasm*, Triple-Negative[Title/Abstract]))) AND (Systematic review)) AND (Meta-Analysis) |
| **Embase** | (('immune checkpoint inhibitor'/exp OR 'checkpoint inhibitor*, immune':ti,ab,kw OR 'immune checkpoint blocker*':ti,ab,kw OR 'checkpoint blocker*, immune':ti,ab,kw OR 'immune checkpoint inhibitor*':ti,ab,kw OR 'ctla*4 inhibitor*':ti,ab,kw OR 'cytotoxic t*lymphocyte*associated protein 4 inhibitor*':ti,ab,kw OR 'pd*1 inhibitor*':ti,ab,kw OR 'programmed cell death protein 1 inhibitor*':ti,ab,kw OR 'inhibitor, pd-1':ti,ab,kw OR 'pd 1 inhibitor':ti,ab,kw OR 'immune checkpoint blockade':ti,ab,kw OR 'checkpoint blockade, immune':ti,ab,kw OR 'immune checkpoint inhibition':ti,ab,kw OR 'checkpoint inhibition, immune':ti,ab,kw OR 'pd*l1 inhibitor*':ti,ab,kw OR 'programmed death*ligand 1 inhibitor*':ti,ab,kw OR 'pd*1*pd*l1 blockade':ti,ab,kw OR 'blockade, pd-1-pd-l1':ti,ab,kw OR ('nivolumab'/exp OR 'mdx 1106':ti,ab,kw OR 'opdivo':ti,ab,kw OR 'bms 936558':ti,ab,kw OR 'ono 4538':ti,ab,kw) OR ('pembrolizumab'/exp OR 'mk-3475':ti,ab,kw OR 'keytruda':ti,ab,kw OR 'lambrolizumab':ti,ab,kw OR 'sch-900475':ti,ab,kw) OR ('camrelizumab'/exp OR 'shr 1210':ti,ab,kw OR 'carrelizumab':ti,ab,kw) OR ('atezolizumab'/exp OR 'anti-pdl1':ti,ab,kw OR 'mpdl*3280a':ti,ab,kw OR 'tecentriq':ti,ab,kw OR 'r-g7446':ti,ab,kw) OR ('avelumab'/exp OR 'msb 0010718c':ti,ab,kw OR 'bavencio':ti,ab,kw OR 'msb 0010682':ti,ab,kw) OR ('durvalumab'/exp OR 'medi*4736':ti,ab,kw OR 'imfinzi':ti,ab,kw) OR ('ipilimumab'/exp OR 'anti-ctla-4 mab ipilimumab':ti,ab,kw OR 'anti ctla 4 mab ipilimumab':ti,ab,kw OR 'ipilimumab, anti-ctla-4 mab':ti,ab,kw OR 'mdx 010':ti,ab,kw OR 'mdx-010':ti,ab,kw OR 'mdx010':ti,ab,kw OR 'mdx-ctla-4':ti,ab,kw OR 'mdx ctla 4':ti,ab,kw OR 'yervoy':ti,ab,kw)) AND ('triple negative breast neoplasms'/exp OR 'er negative pr-negative her2*negative breast cancer*':ti,ab,kw OR 'er negative pr*negative her2*negative breast neoplasm*':ti,ab,kw OR 'triple*negative breast cancer':ti,ab,kw OR 'breast cancer*, triple-negative':ti,ab,kw OR 'triple-negative breast cancer*':ti,ab,kw OR 'triple*negative breast neoplasm*':ti,ab,kw OR 'breast neoplasm*, triple-negative':ti,ab,kw) AND ('systematic review'/exp) AND ('meta analysis'/exp) |
| **Web of Science** | (TS=("Immune Checkpoint Inhibitors" OR "Checkpoint Inhibitor* Immune" OR "Immune Checkpoint Blocker*" OR "Checkpoint Blocker* Immune" OR "Immune Checkpoint Inhibitor*" OR "CTLA*4 Inhibitor*" OR "Cytotoxic T*Lymphocyte*Associated Protein 4 Inhibitor*" OR "PD*1 Inhibitor*" OR  "Programmed Cell Death Protein 1 Inhibitor*" OR "Inhibitor PD-1" OR "PD 1 Inhibitor" OR "Immune Checkpoint Blockade" OR "Checkpoint Blockade Immune" OR "Immune Checkpoint Inhibition" OR "Checkpoint Inhibition Immune" OR "PD*L1 Inhibitor*" OR "Programmed Death*Ligand 1 Inhibitor*" OR "PD*1*PD*L1 Blockade" OR "Blockade PD-1-PD-L1" OR "Nivolumab" OR "MDX 1106" OR "Opdivo" OR "BMS 936558" OR "ONO 4538" OR "Pembrolizumab" OR "MK-3475" OR "Keytruda" OR "lambrolizumab" OR "SCH-900475" OR "Camrelizumab" OR "SHR 1210" OR "carrelizumab" OR "Atezolizumab" OR "anti-PDL1" OR "MPDL*3280A" OR "Tecentriq" OR "R-G7446" OR "Avelumab" OR "MSB 0010718C" OR "bavencio" OR "MSB 0010682" OR "Durvalumab" OR "MEDI*4736" OR "Imfinzi" OR "Ipilimumab" OR "Anti-CTLA-4 MAb Ipilimumab" OR "Anti CTLA 4 MAb Ipilimumab" OR "Ipilimumab Anti-CTLA-4 MAb" OR "MDX 010" OR "MDX-010" OR "MDX010" OR "MDX-CTLA-4" OR "MDX CTLA 4" OR "Yervoy")) AND (TS=("Triple Negative Breast Neoplasms" OR "ER Negative PR-Negative HER2*Negative Breast Cancer*" OR "ER Negative PR*Negative HER2*Negative Breast Neoplasm*" OR "Triple*Negative Breast Cancer" OR "Breast Cancer* Triple-Negative" OR "Triple-Negative Breast Cancer*" OR "Triple*Negative Breast Neoplasm*" OR "Breast Neoplasm* Triple-Negative")) AND (TS=("Systematic review" OR "Meta-Analysis" OR "Meta Analysis")) |
