# Supplementary material for: Efficacy and safety analysis of immune checkpoint inhibitors combined with chemotherapy in the treatment of advanced triple-negative breast cancer: an umbrella meta-analysis
Source: Front Oncol. 2026 Jul 21;16:1900242. doi: 10.3389/fonc.2026.1900242 (PMC13433306; doi:10.3389/fonc.2026.1900242)
Supplement: Supplementary file 3 [file Table3.docx]

**Supplementary Materials**

**Supplementary Material 3**

**GRADE classification of quality of evidence of all included meta-analyses**

| **Author** | **Year** | **Type of included studies** | **Risk of**  **bias** | **Inconsistency** | **Indirectness** | **Imprecision** | **Publication**  **bias** | **Magnitude**  **of effect** | **Plausible**  **confounding** | **Dose-response** | **Quality** |
| --- | --- | --- | --- | --- | --- | --- | --- | --- | --- | --- | --- |
|  |  |  |  |  |  |  |  |  |  |  |  |
| Bo-Ya Xiao | 2020 | RCT | Serious | Serious | Not serious | Serious | Undetected | Yes | Yes | No | ⨁◯◯◯ Very low |
| Trujillo Asturias MA | 2024 | RCT | Serious | Serious | Not serious | Not serious | Undetected | No | No | No | ⨁⨁◯◯  Low |
| Khan M | 2023 | RCT | Serious | Not serious | Not serious | Not serious | Undetected | Yes | No | No | ⨁⨁⨁◯  Moderate |
| Yonghui Chen | 2024 | RCT | Serious | Serious | Not serious | Serious | Not serious | No | No | No | ⨁◯◯◯  Very low |
| Zuxiu Wang | 2024 | RCT | Not serious | Serious | Not serious | Not serious | Undetected | Yes | No | No | ⨁⨁⨁◯  Moderate |
| Juan Yang | 2024 | RCT | Not serious | Not serious | Not serious | Not serious | Undetected | Yes | No | No | ⨁⨁⨁⨁  High |
| Qiao Ji | 2021 | RCT | Not serious | Serious | Not serious | Not serious | Undetected | Yes | No | No | ⨁⨁⨁◯  Moderate |
| Ying Wang | 2024 | RCT | Not serious | Serious | Not serious | Not serious | Undetected | Yes | No | No | ⨁⨁⨁◯ Moderate |
| J. Shen | 2025 | RCT | Not serious | Not serious | Not serious | Not serious | Undetected | Yes | No | No | ⨁⨁⨁⨁ High |
| Youran Dai | 2024 | RCT | Not serious | Not serious | Not serious | Not serious | Undetected | Yes | No | No | ⨁⨁⨁⨁ High |
| Sharmni Vishnu | 2022 | RCT | Not serious | Serious | Not serious | Not serious | Undetected | Yes | No | No | ⨁⨁⨁◯  Moderate |
