# Supplementary material for: Efficacy and safety analysis of immune checkpoint inhibitors combined with chemotherapy in the treatment of advanced triple-negative breast cancer: an umbrella meta-analysis
Source: Front Oncol. 2026 Jul 21;16:1900242. doi: 10.3389/fonc.2026.1900242 (PMC13433306; doi:10.3389/fonc.2026.1900242)
Supplement: Supplementary file 4 [file Table4.docx]

**Supplementary Materials**

**Supplementary Material 4**

**Funnel Chart for assessing publication bias**

**Suppl. Fig. 1 Funnel plots for OS and PFS in advanced TNBC treated with ICIs + chemo: (A) OS; (B) PFS.**

**Suppl. Fig. 2 Funnel plots for ORR in advanced TNBC by PD-L1 status: (A) PD-L1-negative (CPS < 10); (B) PD-L1-positive (CPS ≥ 10).**

**Suppl. Fig. 3 Funnel plots for treatment-related AEs in advanced TNBC receiving ICIs + chemo: (A) All-grade; (B) Grade ≥ 3.**

**Suppl. Fig. 4 Funnel plots for irAEs in advanced TNBC treated with ICIs + chemo: (A) Hypothyroidism; (B) Hyperthyroidism; (C) Rash; (D) Pneumonitis; (E) Hepatitis.**
