# Supplementary material for: Efficacy and safety analysis of immune checkpoint inhibitors combined with chemotherapy in the treatment of advanced triple-negative breast cancer: an umbrella meta-analysis
Source: Front Oncol. 2026 Jul 21;16:1900242. doi: 10.3389/fonc.2026.1900242 (PMC13433306; doi:10.3389/fonc.2026.1900242)
Supplement: Supplementary file 6 [file Table6.docx]

**Supplementary Materials**

**Supplementary Material 6**

**Sensitivity Analysis**

**Suppl. Fig. 9 Sensitivity analysis of survival outcomes in advanced TNBC treated with ICIs + chemo: (A) OS; (B) PFS.**

**Suppl. Fig. 10 Sensitivity analysis of ORR in advanced TNBC by PD-L1 status: (A) PD-L1-negative (CPS < 10); (B) PD-L1-positive (CPS ≥ 10).**

**Suppl. Fig. 11 Sensitivity analysis of treatment-related AEs in advanced TNBC receiving ICIs + chemo: (A) All-grade AEs; (B) Grade ≥ 3 AEs.**

**Suppl. Fig. 12 Sensitivity analysis of irAEs in advanced TNBC treated with ICIs + chemo: (A) Hypothyroidism; (B) Hyperthyroidism; (C) Rash; (D) Pneumonitis; (E) Hepatitis.**
